# The Curious Case of Type I IFN and MxA: Tipping the Immune Balance in AIDS

**DOI:** 10.3389/fimmu.2014.00419

**Published:** 2014-09-02

**Authors:** Andrea Kinga Marias Furuya, Hamayun J. Sharifi, Carlos M. C. de Noronha

**Affiliations:** ^1^Albany Medical Center, Center for Immunology and Microbial Disease, Albany, NY, USA

**Keywords:** HIV, SIV, IFN, MxA

Human immunodeficiency virus (HIV) remains a significant public health challenge. According to the World Health Organization, there were approximately 35.3 million people living with HIV/AIDS worldwide in 2012, with Sub-Saharan Africa being the most affected region (1). HIV has claimed 27 million lives. It is estimated that two million people die from HIV/AIDS each year.

Highly active antiretroviral therapies (HAART) now allow HIV-infected individuals to live longer and healthier lives. HAART is, however, burdened with side effects and high costs, and threatened by evolving viral resistance. A hallmark of both HIV and simian immunodeficiency virus (SIV) infection is chronic immune activation together with uncontrollable viremia and lymphocyte apoptosis that progresses to acquired immunodeficiency syndrome (AIDS) (2, 3). The deleterious role for immune activation in HIV infection is supported by animal studies. Although the exact mechanism underlying AIDS resistance of natural hosts for SIV, like sooty mangabey (SM) monkeys, is unknown, reports support the idea that disease progression in non-natural hosts such as rhesus macaques (RM) is mainly due to immune system dysfunction triggered by HIV/SIV infections (4, 5). In non-natural hosts, SIV causes progressive impairment of the immune system characterized by high viremia, CD4^+^ T cell depletion, and loss of T cell function. Continuous CD4^+^ T cell loss eventually leads to AIDS as the regenerative capacity of the immune system gradually decreases despite the excess of homeostatic cytokines (6). This exhaustion and increased T cell apoptosis, a phenomenon seen in pathogenic infections, may be due to direct and indirect killing by the virus. Indirect- or bystander-killing, is the loss of uninfected and abortively infected T cells possibly due to generalized immune activation (7, 8). It is widely accepted that type I interferon-producing (IFN I) plasmacytoid dendritic cells (pDC) play a central role in this generalized immune activation (9–11). On the other hand, natural simian hosts preserve T lymphocyte populations despite high viremia and show attenuated immune activation that favors the maintenance of CD4^+^ T cells; hence, SMs do not develop AIDS (7). Although HIV preferentially infects CD4^+^ T cells and macrophages, efficient binding and infection of pDCs by HIV have been demonstrated and may contribute to AIDS pathogenesis (12, 13).

Plasmacytoid dendritic cells are specialized cells found in blood and lymphoid tissues. The main function of pDCs is to produce IFN in response to bacterial and viral DNA. Following activation, these produce 1000-fold more IFN I than other IFN producing cells (11). Upon HIV infection, pCDs become activated and express CCR7 and CXCR3, migration markers that induce redistribution to the lymph nodes (LN) (14–16). Further, recent reports suggest that HIV-induced pathogenesis occurs mainly in LNs where the IFN I that pDCs produce elevates serum levels during both acute and chronic infections (13, 17–19).

Type I IFNs are powerful cytokines and adaptive immune system modulators. They are produced upon viral infection, replication, and/or the introduction of double-stranded RNA (20). IFNs trigger antiviral activity and induce the maturation of effector T cells. Therefore, an interesting immunoregulatory role for type I IFN is in lymphocyte activation during viral infection (21). There is, however, evidence that this otherwise beneficial interferon can become detrimental to the host during chronic HIV infection. Persistent levels of IFN I induce apoptosis in both HIV-infected CD4^+^ T cells and in those that do not become productively infected (bystander-killing) leading to accelerated depletion of CD4^+^ T cells during pathogenic infection (22). This effect is due to type I IFN triggered apoptosis of uninfected CD4^+^ T cells via TNF-related apoptosis inducing ligand (TRAIL) (17). Originally, SM, which are natural SIV hosts, were thought to have reduced immune system activation during both acute and chronic SIV infections. Moreover, SM pDCs produce less IFN I *ex vivo* in response to SIV, which leads to less immune activation during chronic infection (23). Other works, however, have shown that natural hosts exhibit an initially strong, but rapidly controlled, IFN I response (24–26). Therefore, both natural and non-natural hosts mount strong type I IFN responses during the acute stage of infection but only natural hosts suppress the response by the chronic stage, 4–16 months after infection. This downregulation occurs despite sustained high levels of viremia. Unlike natural hosts, non-natural hosts maintain high levels of IFN I production at all times. It is important to note that cells from SIV-infected natural hosts can be repeatedly stimulated *in vitro* to produce type I IFN and to upregulate interferon-stimulated genes (ISG) during the chronic stage of SIV infections suggesting that these cells are neither more refractory nor resistant to re-stimulation (26). Therefore, the downmodulation of IFN I in natural hosts is likely due to negative control mechanisms. The exact mechanism underlying the downregulation of IFN I is, however, not fully understood. The regulatory complexity of the IFN pathway and its overall effects on target cells make pinpointing a resolution mechanism challenging. A role for immunomodulatory proteins, negative regulation of IFN responses, and other mechanisms have been suggested [reviewed in Ref. (2)]. Efforts have been made to discover the IFN I downregulation pathway by examining the regulation of ISGs as well as immunosuppressive genes (24, 26). One common denominator is the myxovirus resistance protein (MxA or Mx1) gene. This finding was supported by a recent clinical study in which Chang et al. found that persistent higher expression of type I IFN and ISGs, including MxA, may explain, at least in part, the increased immune activation and more rapid disease progression in females with chronic HIV infections when compared to males with similar viral loads (27). Harris et al. convincingly showed that natural hosts African green monkeys (AGM) and SM begin to downregulate MxA responses by 28 dpi whereas non-natural hosts (RM) maintained high levels of MxA in LN. This work, however, did not reveal a mechanism of IFN I resolution (24). Further, it is unclear whether IFN is directly downregulated or whether downstream signaling negatively regulates IFN. Understanding the mechanism of Mx regulation in the transition phase, from acute to chronic infection of natural hosts, should reveal new targets for therapies to block the chronic immune system activation associated with disease progression.

MxA is an IFN induced protein expressed in cells like macrophages and hepatocytes. It is best known for its antiviral activity against orthomyxoviruses (28). MxA was first described in 1962 when Lindenmann showed that A2G inbred mice were resistant to doses of mouse-adapted influenza virus that were lethal to other inbred mice. This resistance was dependent on a single dominant locus named Mx1 and was exquisitely specific for orthomyxoviruses (29). Later, it was discovered that Mx1 was the first member of a small gene family and that the spectrum of antiviral activity is in fact much larger than originally thought. Most species have one to three Mx protein isoforms with different antiviral activity depending on their intracellular localization (28). Mouse Mx1 protein is found primarily in the nucleus whereas human MxA is cytoplasmic. Hence, each protein blocks influenza virus at a different stage of the viral replication cycle (30). Of note, Mx2, an interferon-induced protein, has recently been shown to inhibit HIV infection after entry (31, 32). Mx2, however, differs from Mx1 in that Mx2 localizes to the nucleus and its antiviral activity relies on a nuclear localization signal (32).

What are Mx proteins and how do they exert their antiviral activity? Most importantly, how are Mx proteins involved in SIV/HIV infections? Mx proteins belong to the superfamily of GTPases, which includes dynamins, dynamin-like proteins, and mitofusins [described in more detail in Ref. (33)]. These proteins are involved in endocytosis, intracellular vesicle transport, and mitochondria distribution. Mx proteins are mainly characterized by three conserved domains: an N-terminal GTPase domain (GTP binding), a middle domain responsible for interaction with the GTPase effector domain (GED), and the C-terminus GED domain, which recognizes the virus. Two amphipatic α-helices form leucine zippers in the C-terminus. Furthermore, Mx proteins can self-assemble into higher order ring-like structures to form a helical stack (28). The higher order structures may represent a storage form whereas the monomers are likely the active form of MxA (34). Human *MxA* can be induced by IFN or directly by the virus through different pathways. Activation of Mx by IFN involves the Janus kinase/signal transducer and activator of transcription (JAK/STAT) pathway and the formation of an IFN-stimulated gene factor 3 (ISGF3) multimeric complex, which in turn migrates into the nucleus and binds an IFN-stimulated response element (ISRE) upstream of the Mx gene (35, 36). It is not yet clear how viral infection activates Mx but it seems to be independent of ISGF3, involving STAT1 instead and possibly the IFN-regulatory factors 1 and 3 (IRF1 and IRF3) (37–39). Mx gene activation is fast. Its protein product is detectable within 4 h (40). It is thus likely that Mx gene activation in response to both virus and IFN through two different pathways is evolutionarily advantageous to the host. Indeed HIV can induce MxA transcription and activate ISGs independently of IFN (41). As mentioned previously, in contrast to non-natural hosts, natural SIV hosts downregulate MxA responses during the transition to chronic infection despite high viremia (24). Moreover with every upregulation/activation, there must be a downregulation of the response in order to maintain homeostasis. Indeed overexpression of MxA is a common pathogenic link in Fanconi Anemia (FA), which consists of a group of at least five autosomal recessive disorders. Overexpression of MxA can lead to cancer susceptibility, apoptosis, bone marrow failure, and abnormal instability in cells (42). One potential mechanism for regulation of Mx gene expression relies on the IRF-1 and IRF-2 proteins. The most probable mediator of Mx induction is IRF-1, which is increased in the presence of virus and after IFN treatment (43, 44). This suggests that the functional synergy between the two independent pathways converges in upregulation of Mx. Additionally, the virus may synergize with IFN to increase Mx levels. IRF-1 induces IRF-2 production to repress ISG transcription. This in turn inhibits IRF-1 function. IRF-1 and IRF-2 share homology in their DNA binding domain; however, IRF-2 has higher affinity for binding and a longer half-life (45). Interestingly, IRF-2 also protects quiescent hematopoietic stem cells (HSC) from type I IFN exhaustion (46). In order to avoid cancer susceptibility, increased apoptosis, bone marrow failure, and HSC depletion, natural SIV hosts apparently evolved to control the IFN responses (Figure 1); however, the exact mechanism is not yet clear. Studying variations in the Mx genes, simultaneously with standardized screening of their antiviral properties in natural and non-natural SIV hosts, could explain why natural hosts can downregulate MxA responses with the onset of the chronic stage of infection. Additionally, mining for polymorphisms in regulatory genes, such as IRF-2, which correlate with enhanced DNA binding or half-life of the activated protein may offer an avenue for designing new therapeutics for controlling the immune system hyperactivation that is associated with AIDS progression.

**Figure 1:**
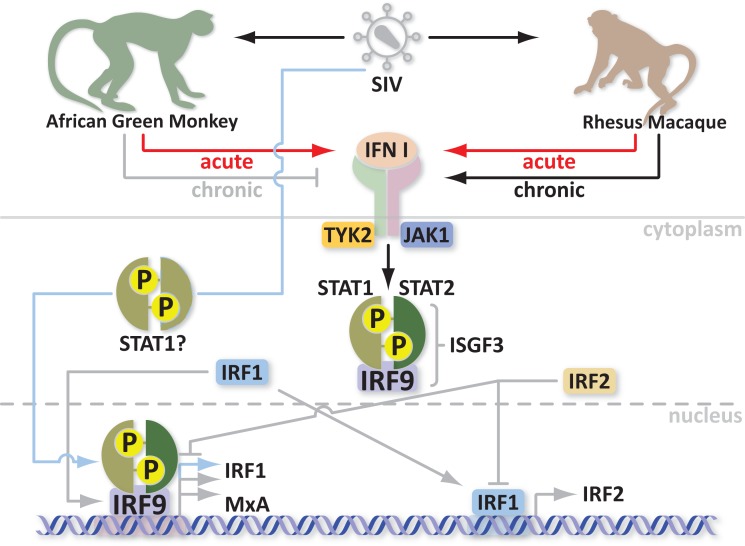
**Schematic of IFN and Mx protein regulation in acute and chronic SIV infection models**.

## Conclusion

Studies in non-human primates have revealed the importance of immune activation in HIV/SIV pathogenesis. Chronic immune activation, associated with CD4^+^ T cell depletion and loss of T cell function, is likely due to increased serum levels of type I IFN. Although type I IFN is necessary to control viral infections, its detrimental effects during the chronic stage of HIV/SIV infection has been well documented. Natural SIV hosts, in sharp contrast to non-natural SIV hosts, control IFN responses with active regulatory mechanisms. Currently, it is not clear how this regulation is achieved on the molecular level. Understanding the basis of active IFN response repression is crucial as these may play an important role in protection against disease progression. Regulation of the type I IFN pathway is very complex making it hard to narrow down the exact factor or factors responsible for repression of this pathway. Evidence supporting a role for MxA genes, warrants studying polymorphisms in MxA genes, which may shed light on the mechanism of active IFN downregulation. Additionally, better understanding of how Mx genes are regulated will further broaden our perspective for understanding the evolution of host–virus interactions.

## Conflict of Interest Statement

The authors declare that the research was conducted in the absence of any commercial or financial relationships that could be construed as a potential conflict of interest.
